# Dyspepsia of Infants

**Published:** 1887-08

**Authors:** 


### ACADEMIE DE MEDICINE, BOURNEVILLE.

(From "*Le Progres Medical.*" No. 21.) Translated by Professor Frank Billings.

PROFESSOR HAYEM read a communication upon the treatment of dyspepsia of infants. He wrote particulary of green diarrhœa and its microbic nature. He finds hydrochloric and lactic acids equally efficacious and salutary. Having observed that when one child was affected with diarrhœa, others in the same crib were quickly affected, he suspected the green stool to be cause. He therefore instituted prophylactic measures; removing soiled linen at once and washing it in a one per centum solution of corrosive sublimate. Since this prophylaxis was instituted green diarrhœa has ceased to exist in the children's wards of the hospital St. Antoine. Professor Hayem and his interne, M. Lesage, have proven the cause of the green color in the stools to be a special bacillus, and that it exists in the intestine. Professor Hayem finds lactic acid the best medicament to neutralize the effects of liquids infected with this bacillus.

